# Expression of cell surface receptors and oxidative metabolism modulation in the clinical continuum of sepsis

**DOI:** 10.1186/cc6801

**Published:** 2008-02-13

**Authors:** Paulo S Martins, Milena KC Brunialti, Leandro SW Martos, Flavia R Machado, Murillo S Assunçao, Sergio Blecher, Reinaldo Salomao

**Affiliations:** 1 Division of Infectious Diseases, Escola Paulista de Medicina, Federal University of Sao Paulo, Brazil; 2 Intensive Care Unit, Hospital Sao Paulo, Federal University of Sao Paulo, Brazil; 3 Intensive Care Unit, Hospital Santa Marcelina, Sao Paulo, Brazil

## Abstract

**Background:**

Infection control depends on adequate microbe recognition and cell activation, yet inflammatory response may lead to organ dysfunction in sepsis. The aims of this study were to evaluate cell activation in the context of sepsis and its correlation with organ dysfunction.

**Methods:**

A total of 41 patients were prospectively enrolled: 14 with sepsis, 12 with severe sepsis and 15 with septic shock. A total of 17 healthy volunteers were included as a control group. Patients were admitted to the Intensive Care Units and Emergency Rooms of Hospital Sao Paulo (Federal University of Sao Paulo) and Hospital Santa Marcelina, Sao Paulo, Brazil. Toll-like receptor (TLR)2, TLR4, CD11b, CD11c and CD66b expression on neutrophil surfaces and oxidative metabolism measured by non-fluorescent dichlorofluorescein (DCFH) oxidation in neutrophils and monocytes, using whole blood, were evaluated using flow cytometry. Organ dysfunction was measured using the sepsis-associated organ failure assessment (SOFA) score.

**Results:**

TLR2 expression on neutrophils was found to be downregulated in septic shock patients compared to healthy volunteers (p = 0.05). No differences were found in CD11b and CD11c expression. CD66b expression was increased in the patient group compared to the control group (p = 0.01). Neutrophil and monocyte oxidative burst was increased in septic patients compared to the control group at baseline and after stimulation with phorbol myristate acetate (PMA), formyl-methionyl-leucyl-phenylalanine (fMLP), lipopolysaccharide (LPS) and *Staphylococcus aureus *(p < 0.001 and p < 0.01, respectively, for neutrophils and monocytes in all tested conditions). A strong correlation was observed between neutrophil and monocyte oxidative metabolism. A SOFA score of 7 discriminated patients between survivors and non-survivors (area under the curve for reactive oxygen species (ROS) was 0.78; p = 0.02). ROS generation in patients with sepsis and septic shock with SOFA scores > 7 was higher than in patients with SOFA scores < 7, both in neutrophils and monocytes. However, oxidative burst in patients with sepsis was as high as in septic shock.

**Conclusion:**

Surface receptors expression on neutrophils may be modulated across the continuum of sepsis, and enhanced or decreased expression may be found depending on the receptor considered. ROS generation is upregulated both in neutrophils and monocytes in septic patients, and it is differently modulated depending on the stage of the disease and the stimuli used.

## Introduction

Severe sepsis and septic shock present with high incidence, morbidity and mortality, and are the most common causes of death in intensive care units [1]. The overall mortality rate is approximately 30%, rising to 50% or more in patients with the more severe syndrome, septic shock, despite recent progress in understanding its pathophysiology and improvements in supportive intensive care [2].

The pathogenesis of sepsis involves a complex interaction between host and infecting microorganism, including bacterial recognition, cell activation and transmigration, phagocytosis and destruction of the pathogen [3-6]. Bacterial recognition and cellular activation are mainly driven by the interaction of pathogen-associated molecular patterns (PAMPs) and the pattern recognition receptors (PRRs), among them the Toll-like receptors (TLRs) [7]. There is some specificity between TLRs and microbial constituents, with TLR4 interacting with lipopolysaccharide (LPS) from Gram-negative bacteria and TLR2 with peptidoglican, lipoteicoic acid from Gram-positive bacteria, lipoproteins from mycobacteria, and others [8,9]. To date, a broad recognition of microbial products by many TLRs has been verified [10]. Current data demonstrate that TLR2 and TLR4 are expressed on the cell surface of neutrophils from healthy volunteers and are upregulated after stimulation by LPS [11] or granulocyte-macrophage colony stimulating factor (GM-CSF) [12]. Moreover, the generation of reactive oxygen species can be triggered through TLRs [13]. Thus, the evaluation of TLRs expression on neutrophils and monocytes in sepsis is of increasing interest.

Once primed by bacterial products and endogenous mediators [14-16], neutrophils adhere to activated cells in inflamed tissues and transmigrate through the endothelial cells to phagocytose and destroy the pathogens [17]. CD66 is a glycoprotein expressed on neutrophils that mediates the interaction with endothelial surfaces triggering a transient activation signal that regulates the adhesive activity of CD11/CD18 [18]. Firm adhesion involves the interaction of leukocyte integrins of the beta 2 subfamily (CD11a, CD11b and CD11c) with endothelial ligand receptors [19]. An increase in the expression of those integrins on neutrophils, in particular CD11b, is considered to be a good marker of cell activation [20].

Invading pathogens are phagocytosed and killed through a potent arsenal of enzymes, cationic proteins, reactive oxygen species (ROS), reactive nitrogen species (RNS), and so on, released into the phagosomes [21,22]. ROS generation in phagocytes is dependent mainly on NADPH-oxidase, a membrane-bound multi-component enzyme complex that once activated works as the electron donor and converts molecular oxygen to its one-electron reduced product, the superoxide anion (O_2_^-^), which is in turn converted to hydrogen peroxide (H_2_O_2_) through a reaction catalyzed by superoxide dismutase. In turn, H_2_O_2 _is a substrate for the generation of other potent oxidants [8,16,23].

ROS generation has been reported to be upregulated in neutrophils from septic patients compared to healthy volunteers upon stimuli with bacterial products and components such as LPS and formyl-methionyl-leucyl-phenylalanine (fMLP) [6,24]. Modulation of ROS production has not been evaluated in monocytes of septic patients; however, they are known to be hyporesponsive to LPS with regard to the production of inflammatory cytokines [25].

In the present study, we evaluated the expression of TLR2, TLR4, CD11b, CD11c and CD66b on the cell surface of neutrophils in patients with sepsis, severe sepsis and septic shock, and compared the findings with those from healthy volunteers. We further evaluated neutrophil and monocyte activation through ROS production, and correlated cell activation and organ dysfunction measured by the sepsis-associated organ failure assessment (SOFA) score [26].

## Materials and methods

### Patients and healthy volunteers

The study population consisted of 41 patients admitted to the Hospital Sao Paulo and Hospital Santa Marcelina, who met at least three clinical criteria for sepsis (14 patients), severe sepsis (12 patients) and septic shock (15 patients) as previously described by Bone *et al. *[27] and reviewed in 2001 by Levy *et al. *[28]. Patients older than 18 years were enrolled within the first 48 h of diagnosis or appearance of the first organ dysfunction or shock in sepsis, severe sepsis and septic shock, respectively. Patients were excluded from the study if they were infected with human immunodeficiency virus, had any neoplasic disease, had received immunosuppressive medications, or if they were participating in any other study protocol. Informed consent guidelines approved by the Ethical Committee of both participant hospitals were required, and obtained from the patients themselves or from the responsible relative in case of unfeasibility of the patient before enrollment in the study.

The mean age and standard deviation of patients was 55.1 ± 20.2 years, and 69% were male. Infection was due to pneumonia (48.7%), urinary tract infection (19.5%), peritonitis (14.6%), bloodstream infection (9.7%) and/or other sources/more than one source of infection (19.5%). Cultures were positive in 10 patients for the following microorganisms: *Staphylococcus aureus, Staphylococcus coagulase negative, Escherichia coli, Providencia *spp, *Klebsiella *sp, *Proteus *sp, *Acinetobacter *and *Enterococcus faecalis*. A total of 60% of the infections were due to Gram-negative microorganisms. The 28-day mortality rate was 24.4% (10/41) and hospital mortality was 36.6% (15/41). Comorbidities were present in 58.5% of the patients, the most found been hypertension, diabetes, congestive heart failure, chronic obstructive pulmonary disease and chronic renal failure. Organ dysfunction was assessed by the SOFA score and was evaluated at day 1 of admission in the severe sepsis and septic shock groups. The median SOFA score was 4.5 in severe sepsis, ranging from 1 to 12 in this group, and 8.5, ranging from 4 to 16, in the septic shock group (p = 0.01). Demographic data, comorbidities, SOFÁ score and outcome of septic patients are expressed in Table 1. Finally, 17 healthy volunteers were included as normal controls. The mean age and standard deviation into this group was 36.4 ± 16.1 years, and 30% were male.

**Table 1:** Demographic data, comorbidities, sepsis-associated organ failure assessment (SOFA) score and outcome from patients

	Sepsis (*n *= 14)	Severe sepsis (*n *= 12)	Septic shock (*n *= 15)
Age, years (mean ± SD)	49.4 ± 19.2	58.4 ± 22.2	57.8 ± 18.9
Male %	78.5	58.3	60
Source of infection, %:			
Pulmonary	42.9	58.3	40
Urinary tract	35.7	8.3	
Peritonitis		25	26.6
Bloodstream	7.1		26.6
Others	14.3	8.3	
Comorbidities	Hypertension	Hypertension	Hypertension
	Diabetes	Diabetes	Diabetes
	CHF	CHF	CHF
	COPD	COPD	COPD
	CRF	CRF	CRF
	CLF	CLF	CLF
			Stroke
Median SOFA score (range)		4.5 (1–12)	8.5 (4–16)
28-day mortality, %	7	41.6	40
Hospital mortality, %	7	50	53.3

CHF, congestive heart failure; CLF, chronic liver failure; COPD, chronic obstructive pulmonary disease; CRF, chronic renal failure; SD, standard deviation.

### Blood sampling

For blood samples, 5 ml of blood were drawn from both healthy volunteers and septic patients into heparin-treated vacuum tubes and 5 ml into ethylenediaminetetraacetic acid (EDTA)-treated tubes (Becton Dickinson, Plymouth, England).

### Flow cytometry analysis

The monoclonal antibodies used were as follows: CD66b-fluorescein isothiocyanate (FITC) clone G1OF5; CD11b-allophycocyanin (APC) clone D12; CD11c-APC clone S-HCL-3 and isotype control mIgG2b-APC clone27-35, obtained from BD Bioscience Pharmingen, San Diego, California, USA, and TLR2-PE clone TL2.1; TLR4-PE clone HTA125 and isotype control mIgG2a-PE clone MOPC-173 were obtained from eBioscience (San Diego, CA, USA).

The expression of cell surface receptors was performed in whole blood, drawn in EDTA tubes. A total of 100 μl of whole blood from patients and controls were stained with 4 μl of CD14-PerCP and 5 μl of CD66-FITC. The tubes were also stained with the following isotypes: 10 μl of mIgG2a-PE and 2 μl of mIgG2b-APC (tube 1); 10 μl of TLR2-PE and 2 μl of CD11c-APC (tube 2) or 20 μl of TLR4-PE and 2 μl of CD11b-APC (tube 3). Samples were incubated with fluorochrome-conjugated monoclonal antibodies for surface staining for 15 min in the dark at room temperature. Red blood cells were ruptured with 2 ml lysis solution (FACS lysing solution, BD Bioscience) for 10 min in the dark at room temperature followed by centrifugation at 2,500 ***g ***for 5 min at 4°C. Then, 2 ml of phosphate-buffered saline (PBS) were added to each tube and centrifuged. Supernatants were discarded and cells were resuspended in 0.3 ml PBS/1% sodium azide.

Event acquisition and analyses were performed using the CellQuest software (BD Bioscience) in a FACSCalibur four-color flow cytometer (BD Bioscience). For each condition 5,000 events were counted in forward- and side-scatter parameters combined with CD14 positive cells. All events were acquired and stored. Neutrophil analyses were performed using forward- and side-scatter parameters combined with CD66b positive and CD14 negative stained cells. The surface receptor expression was measured as the geometric mean fluorescence intensity (GMFI), and results were expressed as the difference between the fluorescence obtained with specific antibodies and isotype controls.

ROS formation was assessed by using 1 ml aliquots of heparinazed whole blood obtained from patients and healthy controls. Oxidative burst was quantified by the addition of 0.06 mM of 2',7'-dichlorofluorescein diacetate (DCFH-DA) in 100 μL of whole blood. DCFH-DA is a stable, non-fluorescent, nonpolar compound that can diffuse through cell membranes. Once inside the cell, the acetyl groups are cleaved by cytosolic enzymes to form the polar non-fluorescent dichlorofluorescein (DCFH), which is rapidly oxidized to highly fluorescent 2',7'-dichlorofluorescein (DCF) in the presence of hydrogen peroxide, thus providing an FL1 fluorescence semi-quantitative assessment of the oxidative metabolism in individual granulocytes and monocytes using flow cytometry.

ROS production was measured constitutively and in response to fMLP (10^5 ^M/ml, Sigma, St Louis, MO), phorbol myristate acetate (PMA, 100 ng/ml, Sigma), LPS of *Salmonella abortus equii *(500 ng/ml, kindly provided by Dr Chris Galanos, Max Planck Institute of Immunobiology, Freiburg, Germany), and to heat-killed *S. aureus *(2.4 × 10^9 ^colony-forming units/ml, ATCC 25923; DIFCO, Detroit, MI, USA). Briefly, the tubes from each sample were incubated in a 37°C shaking water bath for 30 min. Thereafter, 2 ml of 3 mM EDTA (Sigma) was added to each tube followed by centrifugation (250 ***g ***for 10 min at 25°C). Hypotonic lyses in 0.2% saline were performed followed by addition of 1.6% saline and centrifugation (250 ***g ***for 10 min at 25°C). The supernatants were discarded and the pellets were once again incubated with 4 μL of CD14-PerCP at room temperature for 15 min in the dark. Then, 2 ml of PBS was added to each tube followed by centrifugation (250 ***g ***for 10 min at 25°C). The supernatants were discarded and the pellets resuspended in 300 μL of 3 mM EDTA to flow cytometric analysis. For each measurement, cells were acquired based on forward- and side-scatter characteristics and CD14 expression. Neutrophils were gated based on forward- and side-scatter parameters and monocytes by combining these parameters and CD14 expression. ROS generation was expressed as the GMFI in FL1.

### Statistical analysis

Variables were compared between the patient group and healthy volunteers by Mann-Whitney test. Comparisons among healthy volunteers, patients with sepsis, severe sepsis and septic shock were performed by Kruskal-Wallis test. The variables that showed differences among the four groups were compared group to group by Mann-Whitney test. The correlation between variables was analyzed by Pearson correlation test. Area under the curve (ROC) was performed for the analysis of organ dysfunction assessed by the SOFA score. A p value under 0.05 was considered as statistically significant. The results were analyzed using SPSS software (version 13.0; SPSS Inc., Chicago, IL, USA).

## Results

### TLR2 and TLR4 expression on neutrophil surface

**Figure 1:**
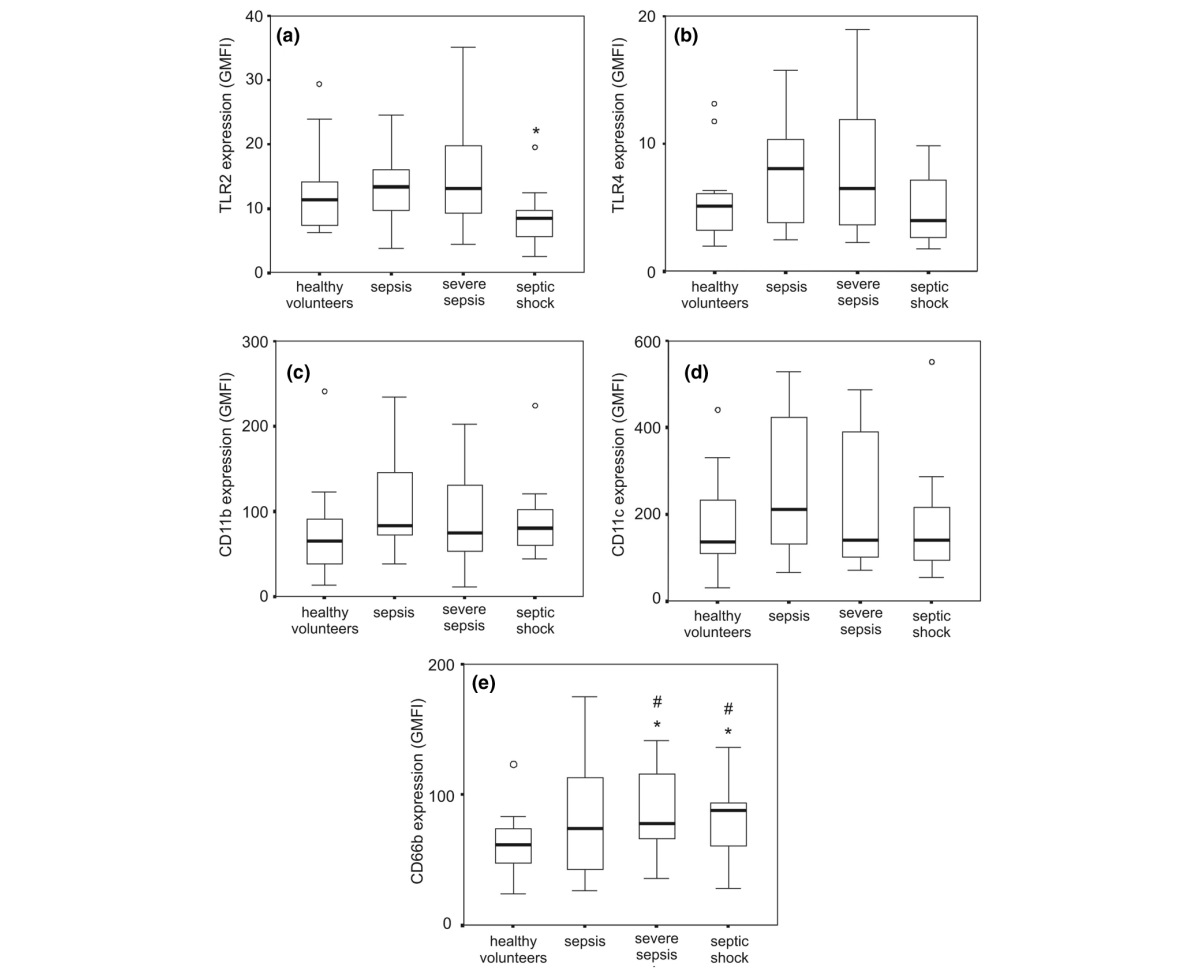
Analysis of the expression of surface receptors on neutrophils in whole blood in healthy volunteers (*n *= 17), patients with sepsis (*n *= 14), severe sepsis (*n *= 12) and septic shock (*n *= 15). Neutrophils were gated based on FSC versus SSC (forward scatter and side scatter) parameters and CD66b+ CD14- stained cells. Surface markers were analyzed in histograms and expressed as the geometric mean fluorescence intensity (GMFI): Toll-like receptor (TLR)2 (A), TLR4 (B), CD11b (C), CD11c (D) and CD66b (E). Values represented are median, range, and quartiles 25–75%. 'o' represents outliers; *p < 0.05 compared to the other groups; #p < 0.05 compared to healthy volunteers (Kruskal-Wallis followed by Mann-Whitney test).

There was no difference in TLR2 expression in the patient group compared to the control group. The comparison among the four groups showed a trend to significance (p = 0.08) with lower TLR2 expression in the septic shock group compared to the control group (p = 0.05), to the sepsis group (p = 0.03) and severe sepsis group (p = 0.04). There was no difference in TLR4 expression among the four groups (p = 0.297) (Figure 1A,B).

### CD11b, CD11c and CD66b expression on neutrophil surface

There were no differences in the expression of CD11b and CD11c among the four groups (p = 0.45 and p = 0.34, respectively) (Figure 1C,D).

The expression of CD66b was significantly higher in the patient group (median GMFI of 84.1 varying from 26 to 175.3) compared to the control group (median of 61.6 varying from 23.9 to 123.1) (p = 0.01). Additionally, the comparison among the four groups showed a higher CD66b expression in the severe sepsis and septic shock groups compared to healthy volunteers (p = 0.02 and p = 0.01, respectively) (Figure 1E).

There was no correlation between the surface expression of neutrophil inflammatory markers and the total white blood cell count or absolute neutrophil count in these patients (data not shown).

### Neutrophil oxidative metabolism

ROS generation was enhanced in the patient group compared to the control group constitutively and after PMA, fMLP, LPS and *S. aureus *stimulation (p < 0.001 in all tested conditions). The median GMFI and range values in the patient group and control group at baseline and after diverse stimuli are shown in Table 2.

**Table 2:** Neutrophil and monocyte ROS generation in septic patients (*n *= 41) and controls (*n *= 17) constitutively and after PMA, fMLP, LPS and *S. aureus *stimulation. Values are expressed in median and ranges of GMFI. p < 0.01 comparing patients and controls in all tested conditions.

	Neutrophil		Monocyte	
	Patients	Controls	Patients	Controls
	
Baseline	55.9 (17 to 167.9)	14.9 (8 to 77)	32.6 (11.7 to 130.5)	8.3 (3.7 to 32.1)
PMA	59.6 (31.7 to 1,630.1)	28.5 (11.2 to 487.4)	44.3 (3.4 to 375.9)	14.5 (5.7 to 63)
fMLP	102.6 (19.5 to 584.3)	10.5 (1.8 to 142)	15.6 (1.3 to 183.4)	5.4 (1.5 to 51.9)
LPS	103.9 (46.2 to 343.5)	18.6 (1.8 to 63.3)	41.2 (1.5 to 116.6)	8.6 (1.4 to 25)
*S. aureus*	419.2 (93.3 to 1,356.2)	48.1 (24.9 to 155)	79.4 (26.4 to 322.9)	15.4 (9.7 to 31.4)

**Figure 2:**
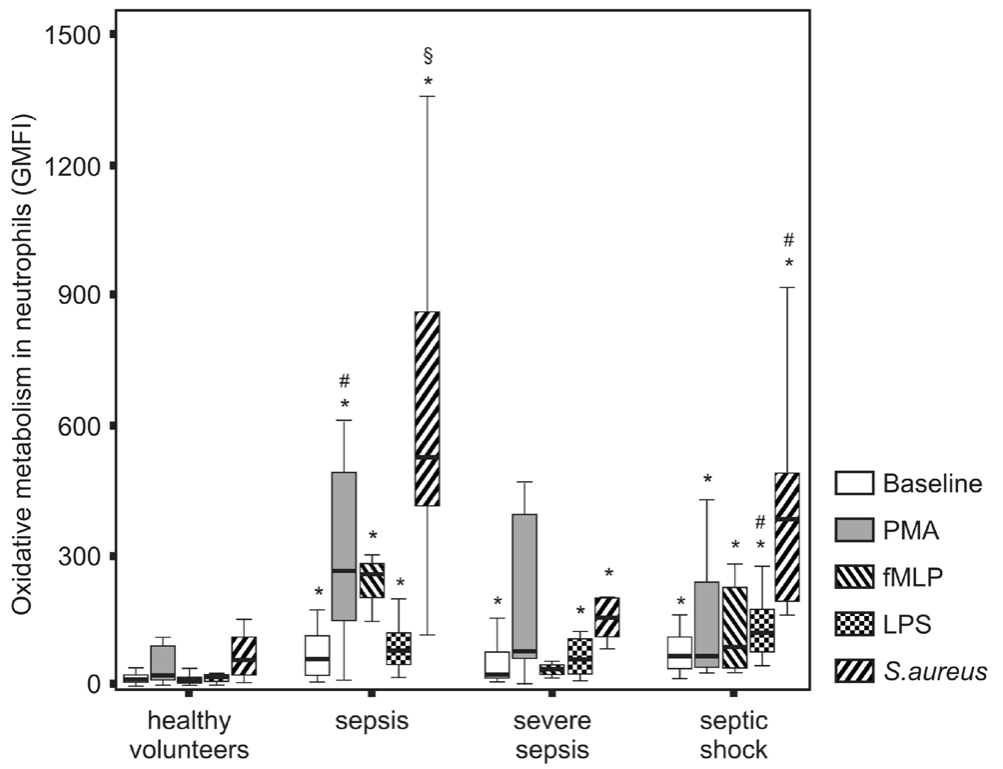
Neutrophil reactive oxygen species (ROS) generation in patients across the continuum of sepsis. ROS generation was measured by 2',7'dichlorofluorescein (DCFH) metabolism. Neutrophils were gated based on FSC versus SSC parameters and the expression of CD14 on cell surface. ROS generation was analyzed in histograms and expressed as the geometric mean fluorescence intensity (GMFI). *p < 0.01 compared to healthy volunteers; #p < 0.05 compared to the severe sepsis group; §p < 0.05 compared to the septic shock group.

The comparison among the four groups showed an increased ROS formation at baseline and after diverse stimuli in all groups of patients compared to healthy volunteers (p < 0.01), except after PMA and fMLP stimuli in severe sepsis (p = 0.15 and p = 0.17, respectively). ROS generation was diminished in severe sepsis compared to sepsis after stimulation with PMA (p = 0.04) and to septic shock after LPS (p = 0.02) and *S. aureus *(p = 0.04). ROS generation was higher in sepsis compared to septic shock after stimulation with *S. aureus *(p = 0.04) (Figure 2).

### Monocyte oxidative metabolism

ROS generation was enhanced in monocytes from septic patients compared to healthy volunteers at baseline and after stimulation with PMA, fMLP, LPS and *S. aureus *(p < 0.01 in all tested conditions). The median GMFI and range in the patient group and in the control group at baseline and after the diverse stimuli are also shown in Table 2.

**Figure 3:**
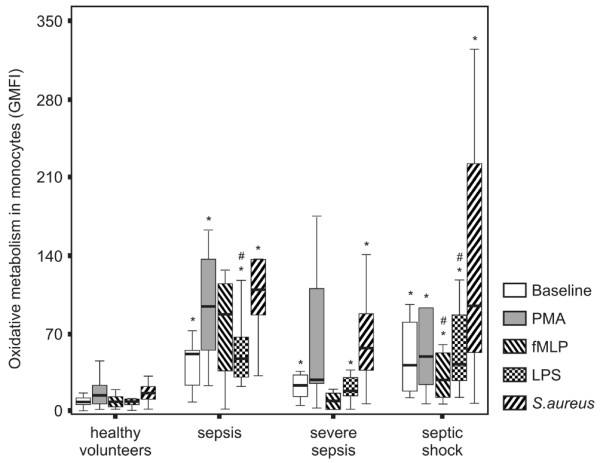
Monocyte reactive oxygen species (ROS) generation in patients across the continuum of sepsis. ROS generation was measured by 2',7'dichlorofluorescein (DCFH) metabolism. Monocytes were gated based on FSC versus SSC parameters and the expression of CD14 on cell surface. ROS generation was analyzed in histograms and expressed as the geometric mean fluorescence intensity (GMFI). *p < 0.01 compared to healthy volunteers; #p < 0.05 compared to the severe sepsis group.

The comparison among the four groups showed an increased ROS formation at baseline and after diverse stimuli in all groups of patients compared to healthy volunteers (p < 0.01), except after fMLP stimulation in sepsis and after PMA and fMLP stimulation in severe sepsis (p = 0.17, p = 0.11 and p = 0.96, respectively). ROS generation was diminished in severe sepsis compared to sepsis after stimulation with LPS (p = 0.01) and to septic shock after fMLP (0.03) and LPS (p = 0.008) (Figure 3).

### Correlation between neutrophil and monocyte oxidative metabolism in septic patients

**Figure 4:**
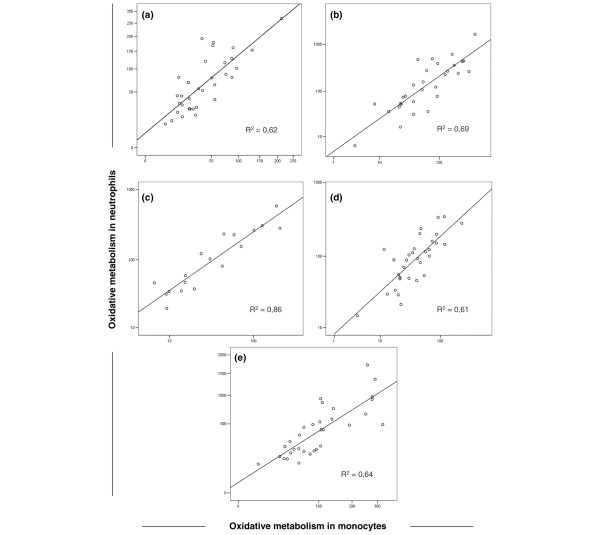
Correlation between neutrophil and monocyte oxidative metabolism in septic patients at baseline (A) and after stimuli with phorbol myristate acetate (PMA) (B), formyl-methionyl-leucyl-phenylalanine (fMLP) (C), lipopolysaccharide (LPS) (D) and *S. aureus *(E).

A strong correlation between neutrophil and monocyte oxidative metabolism in septic patients was found at baseline (R^2 ^= 0.62) and after stimuli with PMA (R^2 ^= 0,69), fMLP (R^2 ^= 0,86), LPS (R^2 ^= 0,61) and *S. aureus *(R^2 ^= 0,64). Results are shown in Figure 4.

### Oxidative metabolism and organ dysfunction

Organ dysfunction was evaluated by the SOFA score in patients with severe sepsis and septic shock (Table 1). A ROC curve was calculated for SOFA score and 28-day mortality. The area under the curve was 0.78 (95% confidence interval (CI) 0.60 to 0.96; p = 0.02). A SOFA score of 7 was the level that discriminated survivors from non-survivors with 72% sensitivity and 70% specificity, and a relative risk (RR) for death of 2.67 (95% CI 0.93 to 7.69; p = 0.01).

**Figure 5:**
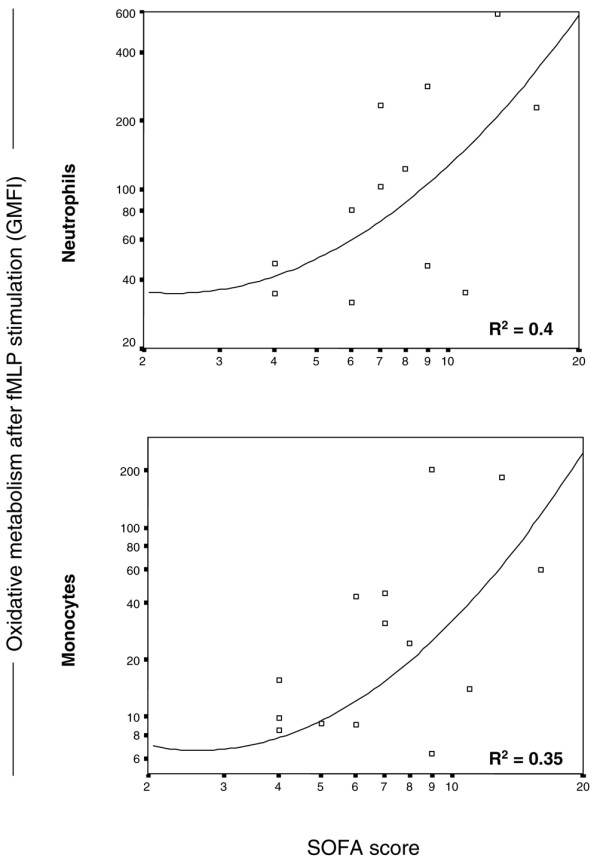
Correlation between formyl-methionyl-leucyl-phenylalanine (fMLP)-induced reactive oxygen species (ROS) generation and sepsis-associated organ failure assessment (SOFA) score in neutrophils and monocytes in patients with severe sepsis and septic shock.

Neutrophil oxidative metabolism was higher in patients with SOFA scores ≥ 7 than with SOFA scores < 7 following fMLP (p = 0.02) and LPS (p = 0.06) stimulation. Similar results were found for monocytes (fMLP, p = 0.02; LPS, p = 0.04). Accordingly, a positive correlation was found between fMLP-induced ROS generation and SOFA score, with increasing values being obtained in patients with SOFA scores ≥ 7 (Figure 5).

## Discussion

In this study we evaluated neutrophil adaptation across the continuum of sepsis. This is a single time point study where the dynamic of sepsis is pointed out by the inclusion of patients at different stages of the disease; sepsis, severe sepsis and septic shock. Data are provided showing modulation of neutrophils functions across this spectrum by assessing the surface cell expression of TLR2 and TLR4, the prototypes of PRRs for Gram-positive and Gram-negative bacteria, CD66b, a selectin with signaling functions [29], and CD11b and CD11c, integrins involved in adhesion with endothelial cells, as well as on signaling for LPS [30]. Neutrophil activity was further assessed by ROS generation, which was associated with the presence and extend of organ dysfunction, assessed by the SOFA score. Furthermore, we evaluated the cell surface expression previously [31] and the oxidative metabolism in monocytes.

In the present study, the expression of cell surface receptors and ROS generation were evaluated in whole blood, with the use of CD66b and CD14 antibodies for immunophenotyping and CD14 antibodies in the assays for ROS generation to better characterize the cell population of neutrophils and monocytes. This approach avoids cellular stimulation, known to occur in cell isolation processes, while providing a good gating for both cell populations.

In clinical studies an enhanced TLR2 and TLR4 expression was observed in leukocytes from septic patients compared to healthy controls [32]. By contrast, we found a decreased expression of TLR2 in patients with septic shock. Expression of TLR2 and TLR4 on monocytes did not differ among healthy volunteers and the groups of septic patients, and are discussed in detail elsewhere [31].

We found an enhanced expression of CD66b in the groups of septic patients that was significantly higher in patients with severe sepsis and septic shock than in healthy volunteers. These results are in agreement with another clinical study where septic patients also showed a higher expression of CD66b on the neutrophil surface compared to healthy controls [33]. An increase in CD66b expression on neutrophils from healthy volunteers following *E. coli *endotoxin infusion was also observed [34], the same occurring in neutrophils isolated from healthy donor blood and pre-stimulated with GM-CSF and tumor necrosis factor alpha (TNFa) [35].

Studies using CD66 as an activation marker in sepsis are still complex; nevertheless, other studies have suggested a broader role for this molecule in neutrophil function, including regulation of integrin-mediated adhesion [36] and potentiation of reactive oxidative species production [37].

In our study, CD11b and CD11c expression did not differ among the groups of patients and controls. The expression of these integrins on neutrophils is clearly induced *ex vivo *upon LPS stimulation [38]. In a clinical setting, Russwurm *et al. *[20] found increased CD11b expression in patients with sepsis and septic shock compared to healthy volunteers. In another assessment of patients with septic shock, an upregulation of neutrophil adhesion measured by CD11b expression was also observed [39]. However, Nakae *et al. *reported decreased expression of CD11b in septic patients compared to patients with trauma not complicated by infection [40]. Even in the case of preserved (our data) or enhanced [20,39] CD11b expression, it is likely that neutrophils will adhere to endothelial cells, but not necessarily the case that they will migrate to the infected site, as reviewed by Brown *et al*. [6].

In this report we confirm that ROS generation at baseline and on different stimuli was higher in septic patients than in healthy volunteers [24]. ROS generation was assessed upon stimuli with PMA, which crosses the cellular membrane and binds to protein kinase C independent of cell receptor interaction, fMLP and LPS (bacterial agents from Gram-positive bacteria and Gram-negative bacteria, which have distinct receptor populations), and *S. aureus *as a heat-killed whole bacteria to induce respiratory burst through bacterial phagocytosis. Patients with sepsis presented ROS generation as high as those with septic shock, with somewhat lower values being observed in patients with severe sepsis. Furthermore, considering the SOFA score from patients with severe sepsis and septic shock, an increased ROS generation was found in patients with SOFA scores higher than 7, the cut-off point that discriminated survivors from non-survivors between our patients.

It may be speculated that early in the disease process a vigorous ROS generation may be desirable and important to restrain the infecting microorganisms. Later in this process, the persistence of increased ROS generation may be deleterious, as indicated by the association of ROS generation and increased SOFA score. Thus, interpretation of ROS generation must consider the clinical setting. In contrast to our results, Kaufmann *et al. *reported an increasing spontaneous hydrogen peroxide production upon fMLP stimulation in patients with rising sepsis severity [41].

ROS production is also a function of monocytes and macrophages and has not been previously evaluated in septic patients. We found, similar to neutrophils, upregulated ROS generation in monocytes in all groups of septic patients. Indeed, a striking positive correlation was found between monocyte and neutrophil ROS production. This upregulated monocyte function contrasts with the downregulation of inflammatory cytokine production found in the same groups of patients with severe sepsis and septic shock reported elsewhere [31] and also with previous reports [42,43]. This discrepancy between decreased cytokine production and enhanced respiratory burst has been recently reported in *in vitro *studies with human and murine macrophages. It has been shown that antimicrobial peptides (AMPs) inhibited TNFa and nitric oxide release by endotoxins, while pre-incubation with AMPs and endotoxin enhanced the respiratory burst [44]. The respiratory burst induced by endotoxin was CD14 and TLR4 dependent, though the AMP-induced respiratory burst was TLR4 independent. The synergistic effect of AMP and endotoxin was a result of a direct effect exerted on ROS generating enzymes, mainly the NADPH oxidase complex [45]. AMPs, endotoxin and other bacterial products may be present in the milieu of circulating cells in septic patients and would support our diverging cytokine and ROS results. This is further evidence that monocyte adaptation in sepsis is not a shutdown process and preserved or even upregulated functions are present, as is clearly shown from the monocyte studies demonstrating up- and downregulated genes in sepsis [46].

## Conclusion

In summary, we show complex neutrophil adaptation in septic patients. Surface receptors expression may be modulated across the continuum of sepsis, and enhanced or decreased expression may be found, depending on the receptor considered and possibly on the stage of the disease. ROS generation is upregulated both in neutrophils and monocytes and its pathophysiology must be interpreted considering the clinical status of septic patients.

## Key messages

Neutrophil surface receptors are modulated across the different stages of sepsis.

ROS generation is enhanced in the neutrophils and monocytes of septic patients.

ROS generation in the neutrophils and monocytes of patients with severe sepsis and septic shock are associated with an increased SOFA score.

ROS production by monocytes in sepsis may be differently regulated from inflammatory cytokines.

There is no clear association between TLR2 and TLR4 expression and ROS generation in neutrophils from septic patients.

## Abbreviations

AMP = antimicrobial peptide; DCFH-DA = 2',7'dichlorofluorescein diacetate; fMLP = formyl-methionyl-leucyl-phenylalanine; GM-CSF = granulocyte-macrophage colony stimulating factor; GMFI = geometric mean fluorescence intensity; LPS = lipopolysaccharide; PAMPs = pathogen associated molecular pattern; PMA = phorbol myristate acetate; PRR = pattern recognition receptor; RNS = reactive nitrogen species; ROS = reactive oxygen species; RR = relative risk; SOFA = sepsis-associated organ failure assessment; TLR = toll-like receptor; TNFα = tumor necrosis factor alpha.

## Competing interests

The authors declare that they have no competing interests.

## Authors' contributions

PSM and MKB participated in the design of the study, laboratory tests, and in the writing of the manuscript. LSWM performed the laboratory tests. FRM, MSA and SB recruited patients and discussed results. RS participated in the design of the study, inclusion of patients, discussion of results, and in the writing of the manuscript. All authors read and approved the manuscript.
